# The Relationship Between Dementia, Race, and Schizophrenia Labeling in Nursing Homes Following the CMS Partnership

**DOI:** 10.1093/geroni/igaa057.071

**Published:** 2020-12-16

**Authors:** Shekinah Fashaw, Theresa Shireman, Ellen McCreedy, Julie Bynum

**Affiliations:** 1 Brown University, Providence, Rhode Island, United States; 2 University of Michigan, Ann Arbor, Michigan, United States

## Abstract

Preliminary research demonstrated an increase in the reporting of schizophrenia diagnoses among nursing home (NH) residents subsequent to the Centers for Medicare & Medicaid Services (CMS) National Partnership to Improve Dementia Care. Given known health disparities and higher antipsychotic use for Black NH residents, we examined how race and dementia influence the rate of schizophrenia diagnoses among NH residents. Using a quasi-experimental study design, we examined changes in schizophrenia reporting among long-stay NH residents’ quarterly and/or yearly Minimum Data Set 3.0 assessments between 2011-2015. Employing a difference-in-difference analysis adjusting for independent variables (e.g. demographics, diabetes, heart conditions, and functional status), we examined the relationship before and after the partnership. There were over 4 million MDS assessments annually. Schizophrenia reporting increased 12.3% in the dementia group as compared to 9.3% in the non-dementia group (p<0.0001). Black residents had a significantly higher likelihood of schizophrenia reporting (4%, p<0.0001). After controlling for covariates, there was a 16.5% increase in schizophrenia reporting for Blacks with dementia and 14.9% increase for non-Blacks with dementia (p<0.0001). There were no racial disparities identified among the non-dementia group after controlling for covariates. NH residents were more likely to have schizophrenia documented on their MDS assessments, and following the partnership, schizophrenia reporting rates increased faster for Blacks with dementia than their non-Black peers and their peers without dementia. Further work is needed to determine if schizophrenia diagnoses are appropriately employed in NH practice, particularly for Black Americans and persons with dementia.

